# *SETX* mutations are a frequent genetic cause of juvenile and adult onset cerebellar ataxia with neuropathy and elevated serum alpha-fetoprotein

**DOI:** 10.1186/1750-1172-8-123

**Published:** 2013-08-14

**Authors:** Lorenzo Nanetti, Simona Cavalieri, Viviana Pensato, Alessandra Erbetta, Davide Pareyson, Marta Panzeri, Giovanna Zorzi, Carlo Antozzi, Isabella Moroni, Cinzia Gellera, Alfredo Brusco, Caterina Mariotti

**Affiliations:** 1 Unit of Genetics of Neurodegenerative and Metabolic Diseases, Fondazione IRCCS Istituto Neurologico “Carlo Besta”, Milan, Italy; 2 SCDU Medical Genetics, A.O. Città della Salute e della Scienza, Turin, Italy; 3 Neuroradiology Department, Fondazione IRCCS Istituto Neurologico “Carlo Besta”, Milan, Italy; 4 Unit of Peripheral and Central Neuropathies, Fondazione IRCCS Istituto Neurologico “Carlo Besta”, Milan, Italy; 5 Child Neurology Department, Fondazione IRCCS Istituto Neurologico “Carlo Besta”, Milan, Italy; 6 Neuroimmunology and Neuromuscular Diseases, Fondazione IRCCS Istituto Neurologico “Carlo Besta”, Milan, Italy; 7 Department of Medical Sciences, University of Turin, Turin, Italy

**Keywords:** Ataxia with Oculomotor Apraxia type 2, AOA2, Alpha-fetoprotein, Recessive ataxias, Ataxia telangiectasia, ATM

## Abstract

**Objectives/background:**

Ataxia with oculomotor apraxia defines a group of genetically distinct recessive ataxias including ataxia-telangectasia (A-T, *ATM* gene), ataxia with oculomotor apraxia type 1 (AOA1, *APTX* gene) and type 2 (AOA2, *SETX* gene). Although, a few unique clinical features differentiate each of these forms, the patients also share common clinical signs, such as the presence of cerebellar atrophy, sensorimotor axonal neuropathy, and elevated alpha-fetoprotein (AFP) serum level.

**Materials and methods:**

We selected 22 Italian patients from 21 families, presenting progressive cerebellar ataxia, axonal neuropathy, and elevated serum AFP. We screened the coding regions of *ATM*, *APTX* and *SETX* genes for point mutations by direct sequencing or DHPLC, and searched genomic rearrangements in *SETX* by MLPA analysis. In selected cases, quantification of ATM and senataxin proteins was performed by Western blot. Clinical, neurophysiological, and neuroimaging data were collected.

**Results:**

Thirteen patients (12 families) carried *SETX* mutations (AOA2, 57%), two were mutated in *ATM* (A-T), and three in *APTX* (AOA1). In three remaining patients, we could not find pathogenic mutations, and in one case we found, in homozygosis, the *SETX* p.K992R polymorphism (population frequency 1-2%). In AOA2 cases, we identified 14 novel and three reported *SETX* mutations. Signs at onset were gait ataxia and facial dyskinesia, and the age ranged between 11 and 18 years. None had obvious oculomotor apraxia at the latest examination (age 14–45 years). The patient carrying the p.K992R *SETX* polymorphism had a phenotype similar to that of the diagnosed AOA2 patients, while the other three undiagnosed subjects had a very late onset and a few distinguishing clinical features.

**Discussion and conclusions:**

We describe a large series of 13 AOA2 Italian patients. The phenotype was consistent with previous descriptions of AOA2, except for a higher frequency of strabism, and for the absence of oculomotor apraxia. In our survey ~60% of juvenile-to-adult cases with cerebellar ataxia, sensorimotor neuropathy and increased AFP are due to mutations in the *SETX* gene, and a smaller percentage to *APTX* and *ATM* gene mutations.

## Background

Oculomotor apraxia (OMA) associated with cerebellar ataxia was first noted by Boder and Sedgwick [1] in patients with ataxia-telangiectasia (A-T). This abnormality of eye movements consists of an impairment in the generation of horizontal saccades with patients performing compensatory head thrusts.

OMA has been described in four genetically distinct forms of inherited neurodegenerative ataxias, classified as autosomal recessive cerebellar ataxias (ARCA) [2]: ataxia-telangectasia (A-T), A-T like disorder (A-TLD), ataxia with oculomotor apraxia type 1 (AOA1) and ataxia with oculomotor apraxia type 2 (AOA2) [3,4].

In addition to eye movement abnormalities, these recessive ataxias also share further clinical, biochemical and molecular features. All are characterized by early onset progressive cerebellar gait and limb ataxia, peripheral sensory neuropathy and cerebellar degeneration [5]. A-T, AOA2, and occasionally AOA1 present elevated serum levels of alpha-fetoprotein (AFP).

The proteins coded by the causative genes for these four ARCAs are all involved in DNA single or double-strand break repair mechanisms [2]. A-T (MIM 208900), the most prevalent of the four with an incidence of 1 in 300,000, is caused by mutation in the *ATM* (A-T mutated) gene encoding a phosphatidylinositol-3 kinase protein. A-T patients lack ATM protein or its kinase activity, and present extra neurological signs, such as immunodeficiency and cancer susceptibility [6]. A-TLD (MIM 604391) is extremely rare, has a milder clinical course than A-T, and is caused by mutations in the human meiotic recombination 11 gene (*hMRE11*) [7]. Patients with A-TLD do not show telangiectasia, no immunodeficiency and have normal AFP serum concentrations [8].

AOA1 (MIM 208920) is characterized by early onset cerebellar ataxia, neuropathy, mental retardation, hypoalbuminemia and hypercholesterolemia. This disease is caused by mutations in the *APTX* gene, which encodes for aprataxin, a nuclear histidine-triad protein involved in DNA single-strand break repair [9]. AOA1 is particularly frequent in Portugal (1:20000) and in Japan [2].

AOA2 (MIM 606002) has a worldwide distribution, and its prevalence is estimated around 1 in 900,000 [4,5]. This form is caused by mutations in the *SETX* gene encoding for a large DNA/RNA helicase protein involved in the defense against DNA damage and in processing RNAs [2,10-12]. *SETX* gene mutations have also been associated with the autosomal dominant juvenile amyotrophic lateral sclerosis type 4 (ALS4) [13], and with the dominant tremor-ataxia syndrome [14]. In this study, we performed a mutational screening of *ATM*, *APTX*, *SETX* in a selected cohort of twenty-two Italian patients presenting cerebellar ataxia, sensorimotor axonal neuropathy, and elevated AFP. Differential diagnosis among cases with this form of degenerative cerebellar ataxia and increased AFP is discussed.

## Patients and methods

### Patients

We selected a cohort of Italian subjects admitted at our clinic from 2002 to 2012. Inclusion criteria were the presence of progressive cerebellar ataxia, axonal neuropathy, and elevated serum concentrations of AFP (≥ 7 ng/ml) [15]. A total of 22 patients (9 men, 13 women), from 21 families, were included. Age at examination was 37.2 ± 14.3 years (mean ± S.D; range 14–71), and age at onset was 18.6 ± 15.3 (mean ± S.D; range 3–67), assumed on the basis of the first symptom noticed by the patient and/or relatives. Clinical assessment included standard neurological examination and administration of the Scale for the Assessment and Rating of Ataxia (SARA) [16]. Electromyography (EMG), nerve conduction studies, and brain MRI were performed in all, whereas 14/22 performed motor, somatosensory, visual, retinal, and auditory evoked potentials (SEP, VEP, ERG, BAEP), electroencephalogram (EEG), and ocular fundus examinations. Peripheral blood samples were obtained for DNA genetic tests and plasma evaluation of creatine kinase (CK), albumin, and cholesterol. Written informed consents for DNA analyses were obtained from all patients and unaffected family members included in the study.

### Genetic analyses

Total genomic DNA was extracted according to a standard phenol-chloroform protocol. We excluded triplet expansions in the genes causing Friedreich ataxia (*FXN*), spinocerebellar ataxia type 1 and 2 (*ATXN1* and *ATXN2*), and point mutations in the genes associated with ataxia with vitamin E deficiency (*TTPA*) and AOA1 (*APTX*) [17]. *ATM* mutation screening was performed as previously described [18].

### *SETX* mutation screening

Initial genetic analyses included Short Tandem Repeats (STR) analysis in the 9q34 region to discriminate homozygous and heterozygous individuals (protocol available upon request). The *SETX* gene coding region was divided into 24 amplimers corresponding to exons 3–26, except for exon 10 that was further amplified in 18 overlapping fragments (reference sequence accession number: NM_015046.5). Amplicons were run on DHPLC (Transgenomic WAVE System). In STR-homozygous subjects PCR amplification was performed by mixing an equal amount of a healthy individual genomic DNA. A normal control profile was always compared with that of a patient. PCR products showing a shift of the DHPLC peak were directly sequenced using the BIG-Dye cycle sequencing kit and an ABI Prism 3130 XL Avant automatic sequencer (Applera, Foster City, CA, USA).

### Multiplex ligation-dependent probe amplification (MLPA) analysis

A total of 100 ng of genomic DNA was used as starting material for the SALSA MLPA kit P316-B2 for Recessive Ataxias available from MRC Holland (http://www.mrc-holland.com). The kit contained probes for each exon of the *SETX* gene (together with exons for *APTX* and *FXN* genes), as well as control probes for other unrelated genes. PCR products were mixed with 0.2 μl of ROX-500 labeled internal size standard, separated on an ABI Prism 3130 XL Avant automatic sequencer (Applera, Foster City, CA, USA), and analyzed using the GeneScan software ver.3.1. Raw data were interpreted using the Coffalyzer software (available from MRC-Holland). A range from 1 ± 0.2 was considered as a normal exon dosage; a deletion was between 0.3–0.7; a duplication was above 1.3. When intermediate values were found the MLPA analysis was repeated.

### Western-blot analyses

Nuclear protein lysates (20 μg) extracted from lymphoblastoid cell lines, were separated on a 4-12% precast SDS polyacrilamide gel (Biorad, Hercules,CA) and electrotransferred onto nitrocellulose membrane. Membranes were blocked with 5% milk-TBS-0.1% Tween20 and incubated overnight at 4°C with anti-ATM antibody (ab17995, Abcam Cambridge, UK) at 1:5,000 dilution, or with anti-SETX antibody (ab56984, Abcam) at 1:1,000 dilution. As loading control an anti-Vinculin antibody (cat. AB6039, Millipore, Temecula, CA, USA) at 1:5,000 dilution was used. Images were acquired and quantified using a Chemidoc apparatus and ImageLab software (BioRad).

## Results

### Genetic findings

Mutation screening allowed the identification of gene variants in of 17/21 index cases (81%): twelve were diagnosed as AOA2 patients (57%), three AOA1 patients, (14%), and two A-T patients (9.5%).

Three patients were negative for pathogenic mutations in all the tested genes (*SETX*, *ATM*, *APTX*, *FXN*, *ATXN 1*–*2*, *TTPA*), and one carried the *SETX* p.K992R variant, previously reported as a rare polymorphism (p.K992R) [19,20].

**Figure 1:**
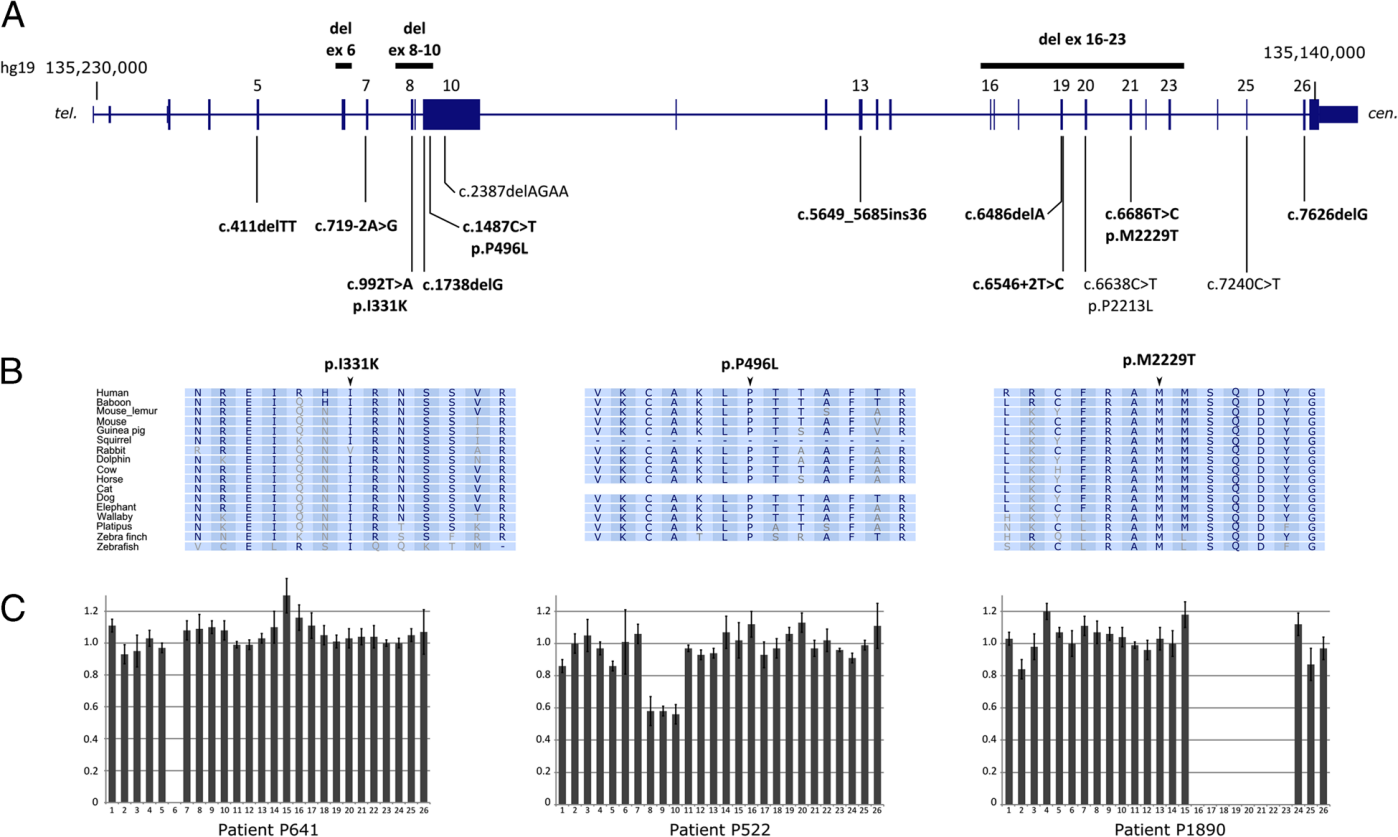
***SETX *gene mutations. (A)** Schematic representation of the *SETX* genomic region between positions ~135,140,000 and ~135,230,000 on chromosome 9 (cen.: centromere; tel.: telomere). Exons are indicated by vertical bars, whose width is proportional to base pair extension. The identified mutations are reported above the region for deletions, and below for point mutations. Novel mutations are in bold. **(B)** Evolutionary conservation of the three novel amino acid changes identified, and flanking protein sequence: p.I133K, p.P496L, p.M2229T. Alignments were obtained from the UCSC genome browser (Conservation Track, http://genome.ucsc.edu/). **(C)** MLPA results in patients P641, P522, P1890, showed a homozygous exon 6 deletion, a heterozygous exon 8–10 deletion and an homozygous exon 16–23 deletion, respectively. In each graph, the abscissa indicates the exon probe, and the ordinates the normalized relative amount of each exon. In case of a homozygous deletion the value reached zero, whereas an heterozygous deletion was suggested by a value in the range 0.4-0.6.

*SETX* gene variants are summarized in Table 1: 14 were novel and 3 mutations (p.R2414X, and p.P2213L, and p.K796fsX15) were previously described in AOA2 patients [10,15]. Six patients were homozygotes. Most of the novel variants were clearly loss-of-function, including nine truncating changes (1 splice site change, 4 small deletions, 4 large deletions). Other variants maintained the reading frame: in one case a splice site mutation caused the in-frame skipping of exon 7; in a second case an insertion of 36-bp in exon 13 included 12 amino acids in-frame. Finally, three novel missense changes were found in compound heterozygous subjects (p.I331L, p.P496L, and p.M2229T) (Table 1). None was reported as SNPs (according to HGMD, NCBI–dbSNP132ver, and Exome Variant Server databases), and involved amino acids highly conserved in Vertebrates (Figure 1). *In silico* analyses using PolyPhen-2 and SIFT predicted the mutations as probably damaging: scores for p.I331K were 0.993 (HumDiv) and 0.909 (HumVar); for p.P496L scores were 1.000 (HumDiv) and 0.999 (HumVar), and for a p.M2229T 0.993 (HumDiv) and 0.914 (HumVar).

**Table 1:** ***SETX*gene mutations in Italian AOA2 patients**

**Patient code**	**Nucleotide changes**	**Exon**	**mRNA or protein change**	**Genetic status**	**Reference**
P815	c.411delTT	5	p.L137LfsX14	Homozygous	This study
P641	c.498-?_718+?del	6	Out of frame ex 6 deletion	Homozygous	This study
P390	c.5649_5685ins36bp	13	p.S1882_L1883ins12	Homozygous	This study
D1642	c.6546+2T>C	19	p.G2184SfsX9	Homozygous	This study
P521	c.6638C>T	20	p.P2213L	Homozygous	Moreira, 2004
P1890	Large deletion Exons16-23	16-23	Predicted ex 16-23 skipping	Homozygous	This study
A317	c.719-2A>G c.6486delA	7	In frame ex 7 skipping p.L2162LfsX42	Compound Heterozygous	This study
		19			
P522	c.992T>A	8	**p.I331K**	Compound Heterozygous	This study
	c.838-?_5724+?del	8-10	Predicted ex 8-10 skipping		
P2426	c.1487C>T	10	**p.P496L**	Compound Heterozygous	This study Anheim, 2009
	c.2387-2390delAGAA	10	p.K796fsX15		
H1207*	c.6686T>C	21	**p.M2229T**	Compound Heterozygous	This study Anheim, 2009
P1277*	c.7240C>T	25	p.R2414X		
P2062	c.7240C>T	25	p.R2414X	Compound Heterozygous	Anheim, 2009 This study
	c.7626delG	26	p.L2542LfsX42		
P657	c.1738delG	10	p.E580KfsX9	Compound Heterozygous	This study
	?	23	out of frame exon 23 skipping		
*P440*	*c.2975A>G*	*10*	*p. K992R*	*Homozygous*	^*§*^*Sequence variant polymorphism*

*SETX* Accession Number: NM_015046.5. In bold the novel missense SETX mutations identified in this study. Asterisk indicate that subjects *P1277* and *H1207* are siblings. §c.2975A>G is a benign genetic variant.

The p.M2229T mutation was found in two affected siblings (H1207 and P1277) in association with a previously described nonsense *SETX* mutation (p.R2414X) (Table 1, Figure 1).

Splicing mutation c.6546+2T>C hits the highly conserved GT of the donor splice site destroying it, and may cause exon 19 skipping or the activation of a new donor splice site within the exon or in the intron 19.

Family segregation analysis could not be performed, however, none of the parents of AOA2 cases showed signs of neurological disorders.

**Figure 2:**
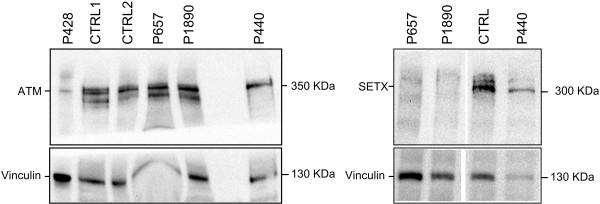
**Western blot analysis of senataxin and ATM proteins.** ATM and senataxin were quantified by Western blot in lymphoblastoid cell line lysates. Vinculin protein quantification was analysed as an internal control for the total protein amount used in the electrophoresis. In the left panel, patient P428 showed a reduced amount of ATM protein, diagnostic of ataxia telangiectasia. In the right panel, senataxin levels were measured in lymphoblasts of P657, P1890 and P440. In the first two patients, the protein was undetectable confirming the diagnosis of AOA2. In patient P440, homozygous carrier of the p.K992R *SETX* variant, we observed low amounts of both the senataxin and the control vinculin protein, compared with the loading control. This finding indicates a relative normal amount of the senataxin protein, and does not support the diagnosis of AOA2 (see Discussion).

In patient P657, Western blot analysis demonstrated the absence of the senataxin protein (Figure 2). We identified a single base pair deletion causing a frameshift, and a large deletion involving at least exon 23, whose boundaries could not be precisely determined.

In patient P440, Western blot analysis showed a normal level of senataxin protein in lymphocytes (Figure 2), and no point variations or genomic rearrangements could be detected except for the rare *SETX* polymorphism c.2975A>G. This changes the lysine at position 992 into an arginine; it was predicted to be benign by *in silico* analysis (PolyPhen-2, HumDiv score 0.034), and it was previously reported as a rare polymorphism, present in 1.6% of the European-American alleles (Exome Variant Server). The variant was found in heterozygosis in the parents and in one unaffected sibling.

One of the two A-T patients carried a novel large *ATM* gene deletion (c.2838+2156del18kb) and a reported missense mutation (c.5471T>C, p.L1824R) [16]. The other patient, third child of consanguineous parents, showed an almost absent ATM protein by Western blot, confirming the diagnosis (Figure 2, patient P428). DHPLC/MLPA failed to reveal pathogenic mutations.

Clinics and gene mutations in the three AOA1 patients were previously described [17].

### Clinics, neuroradiology and biochemical analyses

Clinical and biochemical findings in the 13 patients carrying *SETX* mutations are summarized in Table 2 (mean age at examination: 31.2 ± 10 years). All presented with gait difficulties during the second decade of life (range 11–18 years). In two, mild choreic movements and strabism were noticed before gait abnormalities at the age of 5 and 7 years (Table 2, patients P815 and P2062). Mean age at examination was 31.2 ± 10 years. Eight of the 13 patients were wheelchair-bound, after a disease duration ranging from 7 to 28 (mean 13.6 years). Mean SARA score was 17.9 ± 4.3 (range 9–25). All patients had a progressive ataxic syndrome with nystagmus, slow saccades, dysarthria, gait unsteadiness, dysmetria, and absent deep tendon reflexes at lower limbs. Twelve out of 13 patients had *pes cavus*, 8 distal limb amyotrophy and muscular weakness, 5 Babinski sign, 5 strabism and 3 diplopia. None had a clear oculomotor apraxia, or cognitive impairment. Ocular *fundus* examination was pathological for patient P641 only, who presented a left optic nerve lesion of probable heteroplastic origin.

**Table 2:** **Neurological and biochemical characteristics of patients with*SETX*genetic mutations**

***Patients***	**1 P815**	**2 P641**	**3 P390**	**4 D1642**	**5 P521**	**6 P1890**	**7 A317**	**8 P522**	**9 P2426**	10* **H1207**	11* **P1277**	**12 P2062**	***13 P657***	*P440*
***Sex***/***age***	F/24	M/47	M/45	F/27	F/41	F/21	M/36	F/36	F/14	F/34	M/29	M/20	*F*/*32*	*F*/*33*
***Age at onset of gait ataxia***	14	11	18	18	18	14	14	15	11	17	18	13	*18*	*15*
***Disease duration***	10	36	27	9	23	7	22	21	3	17	11	7	*14*	*18*
***Interval onset***-***wheelchair*** (***years***	na	28	20	9	12	na	10	12	na	11	7	na	*na*	*11*
***Initial symptom***^***2***^	CH (7yrs)	GA	GA	CH/GA	GA	GA	GA	GA	CH/GA	GA	GA	CH (5yrs)	*GA*	*GA*
***Phenotype***														
*OMA*	-	-	-	-	-	-	-	-	-	-	-	-	-	-
*Slow saccades*	+	+	+	+	+	+	+	+	na	+	+	+	+	+
*Strabism*	-	+	-	-	-	-	-	+	+	-	-	+	+	-
*Distal weakness*	+	+	+	+	+	+	+	+	-	-	-	-	+	+
*DTR*	Abs	Abs	Abs	Abs	Abs	Abs	Abs	Abs	Abs^§^	Abs	Abs	Abs	*Abs*	*Abs*
*Babinski sign*	+	-	-	+	-	-	-	-	-	-	-	-	-	-
*Pes cavus*	+	+	+	+	+	-	+	+	-	+	+	+	+	+
***SARA score***	15	25	20	18	21	12	21	20	9	20	19	14	*18*	*23*
***Neurophysiology***														
*Peripheral neuropathy*	+	+	+	+	+	+	+	+	+	+	+	+	+	+
*Denervation at EMG*	no	+	no	no	no	no	+	+	no	+	+	no	+	+
***Brain MRI***														
*Vermian Atrophy*	++	+++	++	++	++	++	++	+++	++	++	++	++	+++	+
*Brainstem atrophy*	-	+	++	-	+	-	+	+	-	no	no	no	no	no
***Biochemistry***														
*AFP*, *ng*/*ml*	34.5	17.1	32.8	34.0	26.6	19.9	36.2	145	15.4	28.5	54.2	19.4	37.7	*27*.*8*
*Cholesterol*, *mg*/*dl*	163	215	140	202	145	203	242	158	na	194	166	142	238	*240*
*CK*, *U*/*L*	66	671	52	89	51	237	178	42	na	75	209	167	63	*37*
***Mutation Type***^***3***^ Allele 1 Allele 2	StopStop	DelDel	StopStop	StopStop	Miss.Miss.	DelDel	DelStop	MissDel		Miss Stop	Miss.Stop	StopStop	Stop??	*Miss*.*Homoz*.*Variant*

^(*)^= these patients were siblings; §= DTR absent only at lower limbs; ^2^Initial Symptoms: *GA* gait ataxia, *CH* choreoathetosis, in parenthesis age in years; *na* not available, *OMA* oculomotor apraxia, *DTR* deep tendon reflexes, *Abs* absent, *SARA* Scale for the Assessment and Rating of Ataxia, *EMG* electromyography, *AFP* alpha-fetoprotein, *CK* creatine kinase (in bold are indicated the values above normal range); + = mild; ++ = moderate; +++ = severe. ^3^Mutation types: Stop mutation, Deletion, Missense mutation (see Table 1).

At the latest clinical evaluation, serum AFP was elevated in all cases (17 to 145 ng/ml, normal value < 7 ng/ml). In two cases (P2426 and P2062), AFP levels were within normal range at the first clinical evaluation performed at 11 and 15 years.

Five out of 12 patients presented borderline or mildly elevated cholesterol levels (202–242 mg/dl), 3/13 mild elevation of CK levels (209, 237, and 671 U/L), and 2/10 decreased triglyceride levels (42 and 44 mg/dl). Albumin was normal.

In all patients EMG showed axonal sensorimotor polyneuropathy prevalent at lower limbs. Motor denervation with spontaneous activity was found in 5/10 patients. In all tested patients, SEP demonstrated a marked sensory peripheral damage, while VEP, ERG, BAEP, and EEG were normal.

A 10-years longitudinal neurophysiological evaluation was available for patient P521. At age 18 (after 3 years of disease duration) EMG demonstrated absent sensory potentials and normal motor potentials at lower limbs; at age 28, neurophysiologic evaluation revealed an axonal peripheral neuropathy at lower limbs involving both the sensory and motor axons.

**Figure 3:**
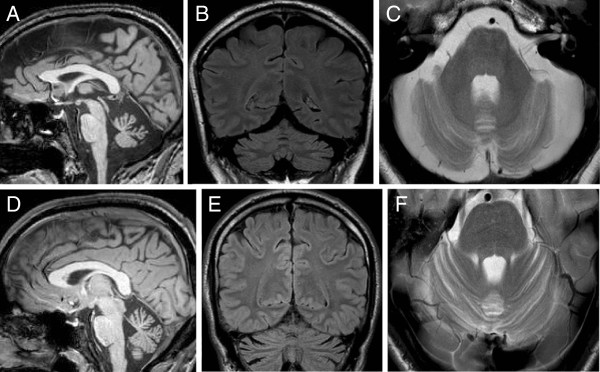
**Brain MRI in AOA2 patients.** Panels **A**-**C** show brain MRI images of patient the P641 with 36-year disease duration: **(A)** Mid-sagittal T1-weighted image showing marked atrophy of the vermis, and moderate atrophy of the midbrain; **(B)** Fluid attenuated Inversion Recovery Image (FLAIR), and **(C)** T2-weighted image confirm marked atrophy of the vermis and show atrophy of the cerebellar hemispheres without abnormal signal intensities of the cerebellar cortex. Panels **D**-**F** show an analogous sequence of brain MRI images from a younger AOA2 patient with 7-year disease duration (P2062). Note the moderate atrophy of the vermis and mild atrophy of the anterior portion of the cerebellar hemispheres without abnormal signal intensity. The midbrain was normal.

Brain MRI demonstrated moderate to marked cerebellar atrophy prevalent in the vermis, and mild to moderate brainstem atrophy in 5 patients (Figure 3).

Patient P440, carrying the *SETX* gene variant p.K992R, presented a clinical, biochemical and neuroimaging features completely overlapping those of the other AOA2 patients with *SETX* pathogenic mutations. She started complaining gait ataxia at 15 years, and was confined to wheelchair 11 years later. She also had sensorimotor peripheral neuropathy, cerebellar atrophy at the vermis, elevated serum AFP (28 ng/ml), and hypercholesterolemia (240 mg/dl) (Table 2).

The two identified A-T patients had the late-onset variant form previously described [21], and presented a neurologic phenotype mostly overlapping that of AOA2 patients: one presented the first signs of gait instability at 7 yrs. (patient A593) and the other at 14 yrs. (patient P428). At the latest clinical examination (age 41 and 52), they presented severe cerebellar ataxia, peripheral sensory neuropathy, slow saccadic eye movements, and nystagmus, but no ocular motor apraxia. Moreover, no immunodeficiency, ocular teleangiectasias, or other systemic features were observed in both. In patient P428 premature ovarian failure (age 30) was reported. Serum concentrations of AFP were 37 (P428) and 207 ng/ml (A593).

## Discussion

We describe the clinic and molecular study of a large series of 22 Italian patients (21 families) selected for having cerebellar ataxia, peripheral neuropathy and elevated alpha-fetoprotein serum level.

Genetic screening of *ATM* (A-T), *APTX* (AOA1) and *SETX* (AOA2) allowed us to find that the prevalent diagnosis was AOA2 (12/21, 57%), followed by AOA1 (3/21, 14%), and A-T (2/21, 9.5%). Concerning these latter, both A-T patients came to our attention in adulthood (age at first examination were 34 and 29 years), and had an atypical A-T phenotype lacking ocular telangiectasia and immunodeficiency [21].

The three patients with mutations in the *APTX* gene had an age at onset in the first decade and present clinical features overlapping those of the atypical A-T patients, but had much lower levels of serum AFP. AFP is not a useful clinical biomarker for AOA1 disease, but in a few patients may contribute to the phenotypic spectrum [17,22].

*SETX* mutations were mostly recognized in patients with juvenile-adult onset. In our group of AOA2 patients, gait ataxia was the first neurological sign, except for two subjects where choreic movements and strabism were noticed several years before the onset of gait difficulties (P2062 and P815). Clinical findings confirm the presence of cerebellar atrophy and sensorimotor neuropathy in all cases [15,23-25]. Also pyramidal signs (20%), choreoathetosis (7.6%), and the rate of disease progression were similar in our patients in comparison to that described in other AOA2 studies [15]. In fact, the time interval between disease onset and the need for wheelchair was 13.6 years in our series, and 15.3 years in the patients described by Anheim et al. [15]. Unexpectedly, we did not find any AOA2 patient with a clear inability to coordinate eyes/head movements to reach a lateral target, in contrast with the 51% of the cases described by Anheim, et al., while we found a higher frequency of strabism (30% vs 12%) [13]. These results may reflect differences in the disease course, since ocular motor apraxia, and choreathetosis, are more frequently observed at the onset of the disease, while cerebellar signs and increased AFP are detectable at later stages.

Fourteen novel *SETX* mutations were found in our AOA2 cohort, the majority leading to a clear loss of function, including five large genomic deletions (5/24, 21% of the mutations). The identification of large deletions has been already described in *SETX* gene, underlining the importance of MPLA analysis for routine diagnostics [26].

Three novel *SETX* mutations were missense: their pathogenicity was mainly assumed on the basis of bioinformatics. The changed amino acid was highly conserved in Vertebrates, and the missense change was not reported as polymorphism in the dbSNP132.

Four patients of our survey did not achieve a genetic diagnosis. Three of them presented an age at onset in the fifth to seventh decade of life with a slowly progressive cerebellar syndrome, and a mild to marked increase of AFP serum concentration. One patient had AOA2 fully compatible neurologic phenotype, including a juvenile age at onset, and markedly elevated level of serum AFP.

We may speculate that they represent phenocopies due to different acquired or genetically caused diseases. A fourth subject (P440) with an AOA2 fully compatible neurologic phenotype also remained without a genetic confirmation. Age at onset was 15 years, and AFP serum levels were within the range of AOA2 patients (Table 2). In this latter subject we could only detect a rare *SETX* polymorphism (p.K992R) previously reported in two AOA2 patients [19,20]. Data from the literature demonstrated that a patient homozygous for the p.K992R, carried also the p.R2444H mutation, the latter regarded as the disease causing mutation [19]. The second AOA2 subject was found to carry three *SETX* mutations: p.K992R, p.H2197R, and p.H435R. Segregation analysis demonstrated that the mother was heterozygous for the p.K992R and the p.H2197R mutations, and the father was heterozygous for the p.H435R [20]. The arguments against the pathogenic role of this variation are that: both AOA2 patients, the p.K992R was found in association with other *SETX* causing mutations, and this variant was also found in 1-2% of control populations (MAF<1%) [19,20]. Variant p.K992R was also found in ALS4 patients, and its pathogenic effect was excluded because it did not segregate with the disease in available families [27]. It may be hypothesized that p.K992R is in linkage disequilibrium with a *SETX* unidentified mutation, that may be located in regulatory regions of the gene, deep-intronic, of involve genomic rearrangements undetected by MLPA; noteworthy, the large exon 10 of the SETX gene is covered by a single MLPA probe in its 5′ end. In any case, we expect a mutation maintaining protein amounts close to normal levels, as demonstrated by Western blot analysis, alternatively this patient is a phenocopy of AOA2 clinically indistinguishable from our AOA2 patients. To this regard, a homozygous missense mutation in a *PIK3R5* was recently described in four siblings from the same family with an AOA2-like phenotype [28]. Mutation screening for this gene could represent a new diagnostic option for our *SETX*-negative patients.

In conclusion, we suggest that patients with ataxia, neuropathy and elevated alpha-fetoprotein should be screened for *SETX* and *ATM* genes, given that an overlapping phenotype may be present among these different genetic defined entities. In negative cases, genetic screening for *APTX1* mutations can also been considered in phenotypically appropriate individuals. Furthermore, we speculate that other disease genes will be found associated with this phenotype and likely an autosomal recessive transmission.

## Abbreviations

A-T: Ataxia-telangectasia; A-TLD: A-T like disorder; AFP: Alpha-fetoprotein; ALS4: Amyotrophic lateral sclerosis type 4; AOA1: Ataxia with oculomotor apraxia type 1; AOA2: Ataxia with oculomotor apraxia type 2; APTX: Aprataxin gene; ARCA: Autosomal recessive cerebellar ataxias; ATM: A-T mutated gene; ATXN1: Ataxin 1 gene; ATXN2: Ataxin 2 gene; BAEP: Brainstem auditory evoked potential; CK: Creatine kinase; DHPLC: Denaturing high pressure liquid chromatography; EMG: Electromyography; ERG: Electroretinogram; FXN: Frataxin gene; hMRE11: Human meiotic recombination 11 gene; MLPA: Multiplex Ligation-dependent probe amplification; OMA: Oculomotor apraxia; SARA: Scale for the assessment and rating of ataxia; SEP: Somatosensory evoked potential; SETX: Senataxin gene; SNP: Single nucleotide polymorphism; STR: Short tandem repeats; VEP: Visual evoked potential.

## Competing interests

The authors declare no conflict of interest.

## Authors’ contributions

LN, AB, CM, study concept and design; analysis and interpretation of data; acquisition of data; drafting and revising the manuscript for content. SC, VP, AE, CG, acquisition of data; analysis and interpretation of data; drafting the manuscript for content. DP, MP, GZ, CA, IM, acquisition of data, analysis and interpretation of data. All authors read and approved the final manuscript.
